# A novel and robust method for counting components within bio-molecular complexes using fluorescence microscopy and statistical modelling

**DOI:** 10.1038/s41598-022-20506-y

**Published:** 2022-10-14

**Authors:** Sophia F. Mersmann, Emma Johns, Tracer Yong, Will A. McEwan, Leo C. James, Edward A. K. Cohen, Joe Grove

**Affiliations:** 1 Department of Mathematics, Imperial College London, South Kensington Campus, London, SW7 2AZ UK; 2 Division of Infection and Immunity, Institute of Immunity and Transplantation, University College London, Pond Street, London, NW3 2QG UK; 3 Department of Clinical Neurosciences, UK Dementia Research Institute at the University of Cambridge, Cambridge Biomedical Campus, Hills Road, Cambridge, CB2 0AH UK; 4 Laboratory of Molecular Biology, Medical Research Council, Cambridge Biomedical Campus, Francis Crick Avenue, Cambridge, CB2 0QH UK; 5 Sir Michael Stoker Building, Garscube Campus, MRC-University of Glasgow Centre for Virus Research (CVR), Glasgow, G61 1QH Scotland, UK

**Keywords:** Image processing, Virology, Fluorescence imaging, Molecular imaging

## Abstract

Cellular biology occurs through myriad interactions between diverse molecular components, many of which assemble in to specific complexes. Various techniques can provide a qualitative survey of which components are found in a given complex. However, quantitative analysis of the absolute number of molecules within a complex (known as stoichiometry) remains challenging. Here we provide a novel method that combines fluorescence microscopy and statistical modelling to derive accurate molecular counts. We have devised a system in which batches of a given biomolecule are differentially labelled with spectrally distinct fluorescent dyes (label A or B), and mixed such that B-labelled molecules are vastly outnumbered by those with label A. Complexes, containing this component, are then simply scored as either being positive or negative for label B. The frequency of positive complexes is directly related to the stoichiometry of interaction and molecular counts can be inferred by statistical modelling. We demonstrate this method using complexes of Adenovirus particles and monoclonal antibodies, achieving counts that are in excellent agreement with previous estimates. Beyond virology, this approach is readily transferable to other experimental systems and, therefore, provides a powerful tool for quantitative molecular biology.

## Introduction

All cellular processes are driven by coordinated networks of dynamically interacting molecular partners. To successfully function, these components typically need to be assembled into specific complexes or clusters. For example, receptor signalling generally requires the co-location of various sensory, regulatory and stimulatory partners; the precise make-up of these assemblies can tune the nature of the signal and resultant physiological output. Molecular cell biology research has, thus far, largely focused on determining the identity of the components found in a given complex. However, it is becoming clear that the quantity of any given component is also vitally important. Quantifying the number of molecules, or stoichiometry, within an assembly can be used to understand its ultrastructure and, ultimately, to create complete models of entire macromolecular assemblies, as has been demonstrated for HIV capsids and the neurological synapse^1,2^.

There are various approaches to investigate the number of molecules within a given complex; for example calibrated biochemical analysis or cryo-electron microscopy (cryo-EM). However, such methods pose practical and/or technological barriers and, by their very nature, obscure heterogeneity within the sample. Single molecule localisation microscopy (SMLM) modalities, such as STORM and PALM, are potentially powerful techniques for counting^3–8^. Nonetheless, these approaches typically require detailed *a priori* knowledge of the experimental system (for instance, a thorough evaluation of the ‘blinking’ characteristics of the fluorophores^9^) and/or a well understood reference sample with which to calibrate the measurement^8,10^. These steps need to be performed independently for each different experimental model and microscope set up; this creates a high barrier to implementation and can make these methods vulnerable to experimental variation and artefacts.

Here we outline an alternative, and potentially complementary, approach that combines (non-SMLM) fluorescent microscopy and statistical modelling to extract estimates of molecular numbers within a complex. The method requires differential binary labelling of a constituent (i.e. separate batches of a protein of interest are labelled with fluorescent dye A or B); by appropriate mixing of the differentially labelled batches, assembled molecular complexes can be simply scored as being positive or negative for a given label. The frequency of positive complexes is then analysed by statistical modelling to extract estimates of stoichiometry. This approach is simple and requires minimal calibration or *a priori* understanding of the experimental system.

We demonstrate the feasibility and accuracy of this approach by studying the stoichiometry of virus-antibody complexes. Adenovirus (AdV) is a non-enveloped DNA virus; its genome is enclosed within a proteinous shell, called a capsid^11^. The major AdV capsid protein is hexon; this assembles into trimeric subunits, that are hexagonal in shape, which in turn arrange to form an icosahedron with 20 triangular faces (a molecular cartoon of the AdV particle is provided in Fig. 3A). The AdV particle has 12 vertices, each of which are occupied by a pentameric subunit (formed of the penton base protein) and a receptor binding ‘spike’ (formed of the fibre protein).

Antibodies (Ab) that bind sites such as the spike can directly neutralise AdV by blocking receptor interactions, therefore preventing the virus particle from entering the cell. However, antibodies targeting the hexon protein (which makes up the majority of the capsid) do not necessarily interfere with the mechanics of virus entry^12,13^. Nonetheless, anti-hexon antibodies can prevent virus infection by recruiting the intracellular antibody-sensor TRIM21, which targets the virus for degradation and activates cell-intrinsic immune responses^14,15^. Anti-hexon monoclonal antibody 9C12 inhibits AdV infection via this mechanism and is used as a prototypical system to investigate TRIM21. The stoichiometries of antibody and TRIM21 recruitment to incoming virus particles remain unclear and are likely to be a determinant of the resulting cellular response.

Previous studies, using alternative techniques, have provided estimates of the stoichiometry of AdV-9C12 complexes. Each AdV particle possesses 720 identical hexon proteins, each of these represents a potential binding site for 9C12. However, the hexon subunits are assembled as trimers, and are arranged in a specific geometry (as described above). Moreover, antibodies are bivalent, therefore each 9C12 molecule has two hexon binding pockets. Consequently, it is highly unlikely that each hexon molecule will be occupied by a single 9C12 molecule (i.e. 720 antibodies per virus particle). Analysis by cryo-EM, immuno-gold EM staining and calibrated fluorescence measurements suggest a true maximum stoichiometry of 100-200 antibodies per particle^16,17^; this maximum is likely determined by the limits to antibody binding and packing enforced by the geometry of the particle. We have applied our counting method to the AdV-9C12 complex and generated estimates that are in good agreement with these previous studies, therefore validating our approach.

## System and methods

### Strategy

**Figure 1:**
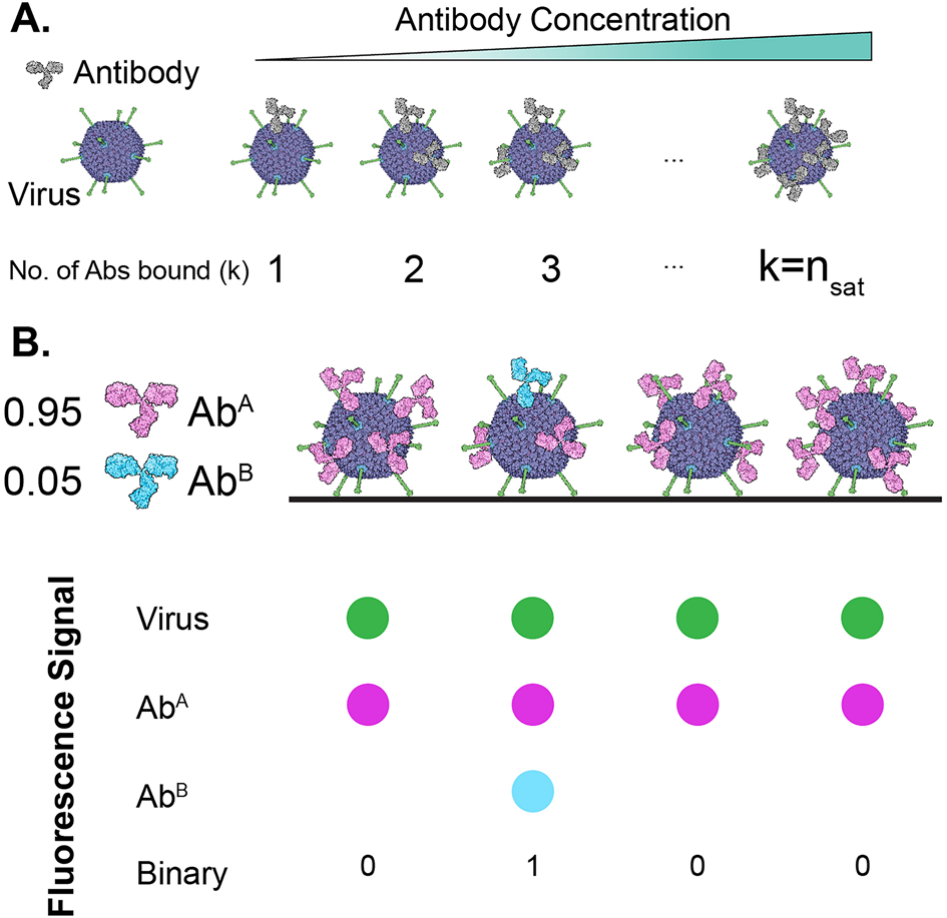
Binary Labelling of AdV-antibody Complexes. (**A**) The ground truth: the number of antibody molecules (*k*) per virus particle increases with antibody concentration up to saturation $(k=n_{\text {sat}})$. (**B**) Extracting truth from data: AdV particles (labelled with a green fluorescent dye) are incubated with a defined mixture of two batches of antibody; one batch has received fluorescent label A (magenta), whilst the second has received label B (blue). When viewed by microscopy, every virus particle has received at least one molecule of Ab^A^, whereas only a minority have received any Ab^B^ and can be scored in a binary fashion. Note, antibody molecules are not drawn to scale.

We used differential binary labelling and statistical modelling to extract estimates of stoichiometry, our strategy is outlined in Fig. 1; note that this approach can be generalised to apply to many other multi-component systems (i.e. how many protein x are found in assembly y?). The hidden truth is the number of antibodies bound to a virus particle; the Ab:virus stoichiometry is expected to increase with antibody concentration until it reaches a saturation point where the maximum number of Abs are bound (Fig. 1A.).

In our method (Fig. 1B), both components (virus and antibody) are fluorescently labelled, however, two separate batches of, the otherwise identical, antibody are given spectrally distinct dyes (resulting in Ab^A^ or Ab^B^). Mixing of the differentially labelled antibody batches at appropriate proportions results in only a minority of virus particles receiving a particular fluorescent dye (B in the example cartoon). Therefore, when imaged, we detect three distinct fluorescent signals: each virus particle can be identified by its reference dye (green in the cartoon example), every virus particle has also received antibodies with dye A (magenta), however, very few particles are positive for dye B (blue) and can be scored as positive or negative in a binary fashion. The frequency of virus particles that are positive for Ab^B^ is a function of the A:B mixing proportion and the stoichiometry of Ab:virus interaction; this relationship between the data and the hidden truth can be modelled.

Consider a single virus to be capable of binding $n_{\text {sat}}$ antibodies at saturation. Under the assumption that antibodies bind to the same virus independently from each other, *K*, the total number of (Ab^A^ and Ab^B^) antibodies bound to a virus, can be modelled as a binomial random variable

$$\begin{aligned} K \sim \text {Bin}(n_{\text {sat}}, p), \end{aligned}$$

where *p* is the probability of an antibody binding to a particular binding site of the virus. If $n_{\text {sat}}$ and *p* are known, then the expected number of antibodies bound to a single virus is simply $\text {E}[K] = n_{\text {sat}} \cdot p.$ However, in most cases $n_{\text {sat}}$ is not known and *p* cannot be expressed easily since it depends not only on the antibody concentration used but also the composition of the virus particle and the geometry of interaction, which can be difficult to obtain. To extrapolate an antibody count it is, therefore, necessary to estimate both, $n_{\text {sat}}$ and *p*.

As described above, our experimental design utilizes antibody labelled with spectrally distinct dyes allowing binary scoring of individual virus particles as positive if they interact with at least one Ab^B^ molecule (Fig. 1). Here, we describe this state as being a Bernoulli random variable *S* that takes the value 1 if the virus is in the positive state, and 0 if it is in the negative state, i.e.

$$\begin{aligned} S \sim \text {Ber}(q), \end{aligned}$$

where *q* is the probability a virus interacts with at least one Ab^B^ molecule.

Since $q = P(S=1) = 1- P(S=0),$ we can derive a closed form for *q* by finding an expression for the probability of a virus not being complexed with any Ab^B^ $P(S = 0)$. To this end, we simply sum over all possible virus-antibody configurations under the constraint of all antibodies being Ab^A^, i.e. a virus could bind zero, one, two, ... up to $n_{\text {sat}}$ Ab^A^ antibodies. The marginal probability of a virus being in a negative state, in respect to Ab^B^, is thus given by

$$\begin{aligned} P(S=0) = \sum _{k=0}^{n_{\text {sat}}} P(S=0 | K=k) P(K=k), \end{aligned}$$

where $P(S = 0 | K = k)$ is the probability that given the virus binds to *k* antibodies, exactly zero of them are Ab^B^. The conditional distribution of *S* given $K=k$ is itself binomial, namely

$$\begin{aligned} S| K = k \sim \text {Bin}(k,f_l) \end{aligned}$$

where $f_l$ is the proportion of antibodies that are Ab^B^. Therefore

$$\begin{aligned} P(S=0|K=k) = (1-f_l)^{k}, \end{aligned}$$

and combining with the probability mass function of *K*, namely

$$\begin{aligned} P(K=k) = \left( {\begin{array}{c}n_{\text {sat}}\\ k\end{array}}\right) p^{k} (1-p)^{n_{\text {sat}}-k}, \end{aligned}$$

gives

$$\begin{aligned} P(S=0) = \sum _{k=0}^{n_{\text {sat}}} (1-f_l)^{k} \left( {\begin{array}{c}n_{\text {sat}}\\ k\end{array}}\right) p^{k} (1-p)^{n_{\text {sat}}-k}. \end{aligned}$$

A closed form for *q* directly follows as

1 $$\begin{aligned} q&= 1 - P(S=0) \nonumber \\&= 1 - \sum _{k=0}^{n_{\text {sat}}} (1-f_l)^{k} \left( {\begin{array}{c}n_{\text {sat}}\\ k\end{array}}\right) p^{k} (1-p)^{n_{\text {sat}}-k}. \end{aligned}$$

Note that here $P(S = 0)$ is expressed in terms of *p* and $n_{\text {sat}}$, and will hereafter be referred to as $P(S = 0; \varvec{\theta })$, where $\varvec{\theta } = (p,n_{\text {sat}})$.

Consider a single experiment (performed at a specific antibody concentration) to describe *V* viruses with states $\mathbf {s} = s_1, ..., s_V$ where $V_+$ of these states are positive, i.e. $V_+$ viruses have been observed to interact with at least one Ab^B^ molecule. Assuming independence among viruses, the likelihood of $\varvec{\theta }$ is then

2 $$\begin{aligned} \mathscr {L}(\varvec{\theta }; \mathbf {s}) = P(\mathbf {s}|\varvec{\theta })= & {} \prod _{i=1}^{V} P(S=s_i; \varvec{\theta })\nonumber \\= & {} V_+ \cdot q + (V-V_+) \cdot (1-q), \end{aligned}$$

where *q* is as given in (1). We are then interested in the posterior distribution of $\varvec{\theta }$ given by the central result of Bayesian statistics,

$$\begin{aligned} \pi (\varvec{\theta } | \mathbf {s}) \propto \mathscr {L}(\varvec{\theta };\mathbf {s}) \cdot \pi (\varvec{\theta }), \end{aligned}$$

where $\pi (\varvec{\theta })$ is a suitable prior for the unknown parameters $\varvec{\theta }$.

In most applications, more than one experiment is performed; consider *m* experiments to be conducted at varying antibody concentrations $c_1, ..., c_m$, producing *m* sets of virus state observations $\mathscr {S} =\{\varvec{s}_1,...,\varvec{s}_m\}$. Parameter inference using the described single-experiment model would entail building *m* independent models, each estimating $p_j$ and $n_{\text {sat},j}$ for experiment *j*. While estimating concentration-specific antibody binding probabilities is desired, inferring multiple $n_{\text {sat}}$ values is unintuitive since $n_{\text {sat}}$ is fixed for a particular virus-antibody pair, i.e. $n_{\text {sat}}$ should be common to all experiments regardless of the antibody concentration used. It is, therefore, preferable to develop a general model accounting for multiple experiments that estimates all $p_1, ..., p_m$ simultaneously while yielding only a single $n_{\text {sat}}$ estimate.

Such a general model contains a single likelihood $\mathscr {L}(\varvec{\theta };\mathscr {S})$, where $\varvec{\theta } = (p_1,...,p_m,n_{\text {sat}})$. This can be expressed as

$$\begin{aligned} \mathscr {L}(\varvec{\theta };\mathscr {S}) = \prod _{j=1}^m \mathscr {L}_j(\varvec{\theta }_j; \mathbf {s}_j) \end{aligned}$$

where $\mathscr {L}_j(\varvec{\theta }_j; \mathbf {s}_j)$ is the likelihood function for $\varvec{\theta }_j = (p_j,n_{\text {sat}})$ as defined in (2). Hence, $m+1$ unknown parameters are estimated; a probability $p_j$ specific to each concentration $c_j$ for $j=1,...,m$, and crucially a single $n_{\text {sat}}$ shared over all experiments. To sample from the posterior distributions Markov Chain Monte Carlo (MCMC) is used, specifically the Metropolis-Hasting algorithm using PyMC^18^. No prior knowledge is incorporated by imposing a beta distribution Beta(1,1) on all $p_j$, $j=1,...,m$, and a uniform distribution with a sufficiently large upper bound, Uniform(0,1000), on $n_{\text {sat}}$. The convergence of MCMC is checked by visual inspection of trace and autocorrelation plots for each parameter. The statistical models used in our analysis are available here: https://github.com/sophiamersmann/molecular-counting, the raw data used for molecular counting can be found here: 10.5281/zenodo.3955142.

### Model verification via simulation

The proposed method makes use of experimental parameters including the proportion of Ab^B^ molecules ($f_l$) and the number of viruses sampled (*V*). Formally, these are not required to comply with specific bounds. However, certain configurations of an experimental setup are not expected to yield data that lead to a sensible model. Using only ’B’ labelled antibodies (Ab^B^) in an experiment (i.e. $f_l$ = 1), for example, would result in a data set with low information content. To explore how different experimental design choices impact the model’s ability to reliably estimate parameters, we analysed simulated data that assumed a range of possible experimental settings.

We simulated a single experiment at a time and, for the purposes of simulation, assumed the number of antibodies bound at saturation to be known. For this we used an upper limit estimate of AdV-9C12 stoichiometry that we previously derived from an alternative method, $n_{\text {sat}} = 205$^16^. In each simulation, the binding probability *p* of an antibody is thus the only parameter estimated. We considered a range of possible experimental settings by varying the total number of viruses sampled in an experiment (V=100−4000) and the proportion of Ab^B^ molecules ($f_l$=0.01−0.9). For an assumed true antibody binding probability $p \in [0.1, 0.99]$, data is simulated by drawing *V* virus states from $S \sim \text {Ber}(q)$ with *q* as described in (1). The antibody binding probability was then blindly estimated using MCMC and convergence checked using the Gelman-Rubin statistic^19^.

**Figure 2:**
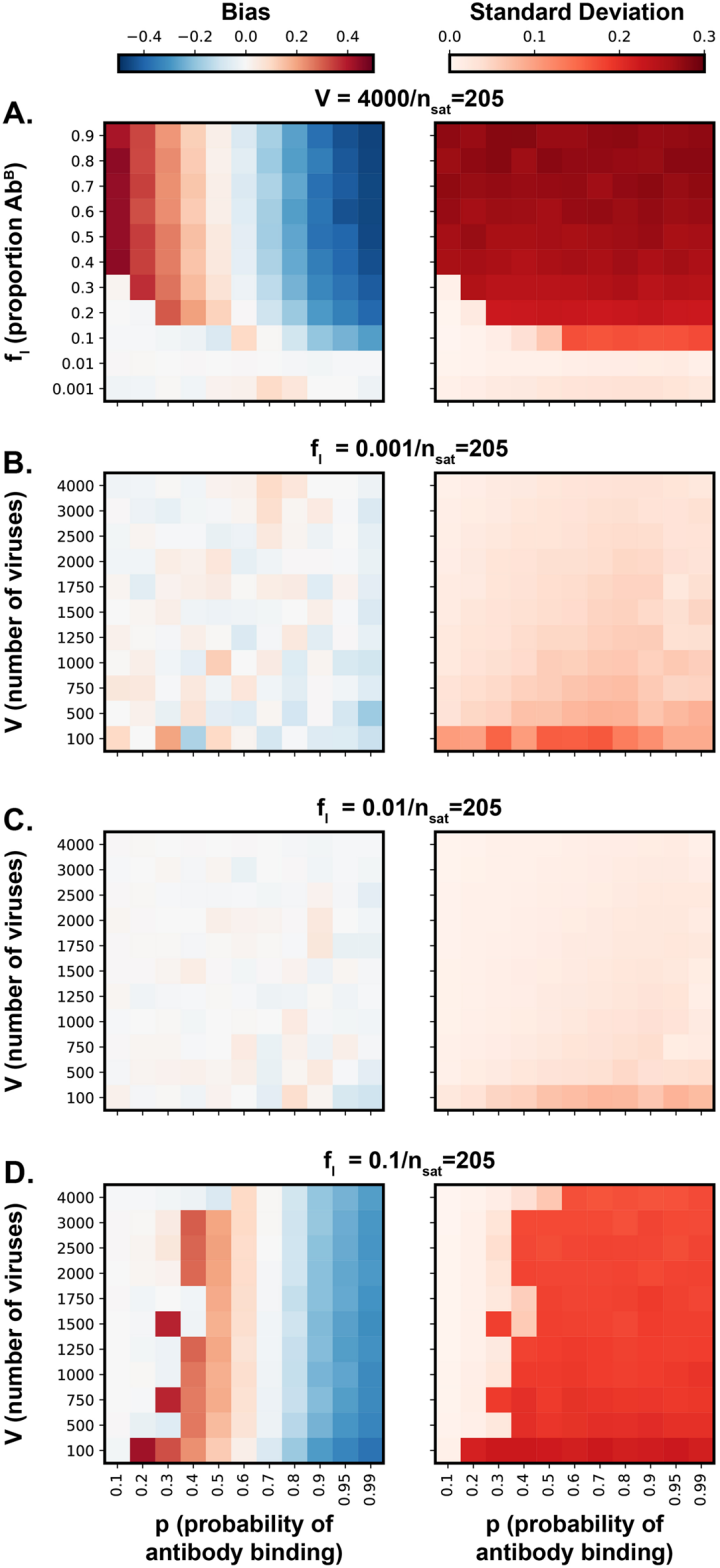
Model verification via simulation. Bias and standard deviation landscapes of the posterior distributions upon increasing antibody binding probabilities, estimated from simulations to explore a range of experimental settings. Here, bias is the mean of the posterior distribution minus the true probability, while standard deviation refers to the standard deviation of the posterior distribution. (**A**) Bias and standard deviation as a function of the true binding probability, *p*, and the proportion of Ab^B^ molecules included, $f_l$. The number of viruses used in an experiment, *V*, is fixed at 4000. (**B–D**) For a fixed value of $f_l$ (0.001, 0.01, and 0.1, respectively), bias and standard deviation are shown as a function of *p* and *V*. In each case $n_{\text {sat}}$=205; this is a reasonable upper limit for AdV-9C12 stoichiometry based on previous estimates.

A total of 1331 simulations were carried out that assess the model’s ability to reliably estimate *p* in various experimental settings. As expected, high proportions of Ab^B^ molecules produced data of low information content, reflected in the model’s inability to accurately estimate *p*, even if the number of viruses used in an experiment was high (Fig. 2A). By contrast, if $f_l$ is less than or equal to 0.1, *p* was estimated with low bias and low variance (Fig. 2A). Simulations also suggested that for low $f_l$, as a rule of thumb, at least 500 viruses per experiment should be sampled (Fig. 2B,C). However, if the proportion of Ab^B^ molecules is 0.1 (or higher), then the proposed model failed to produce a reasonable estimate of *p* for most underlying true values; higher number of viruses seemed to compensate this effect to some extent (Fig. 2D). In summary, these simulations put empirical bounds on experimental parameters and show that, if compliant, the method yields sensible estimates. Notably, we also ran simulations in which $n_{\text {sat}}$ was a free parameter (as opposed to being given a large upper bound), however these calculations proved intractable with MCMC not reaching convergence within a practical amount of time. Therefore, it is recommended that $n_{\text {sat}}$ be given an arbitrarily large upper bound based on an understanding of a given biomolecular assembly. For example the value chosen in our simulations (1000) is >4-fold higher than our simulated ground truth (205) and above the theoretical upper limit of our biolgical system (AdV has a maximum of 720 9C12 binding sites per particle).

## Implementation

### Experimental setup

**Figure 3:**
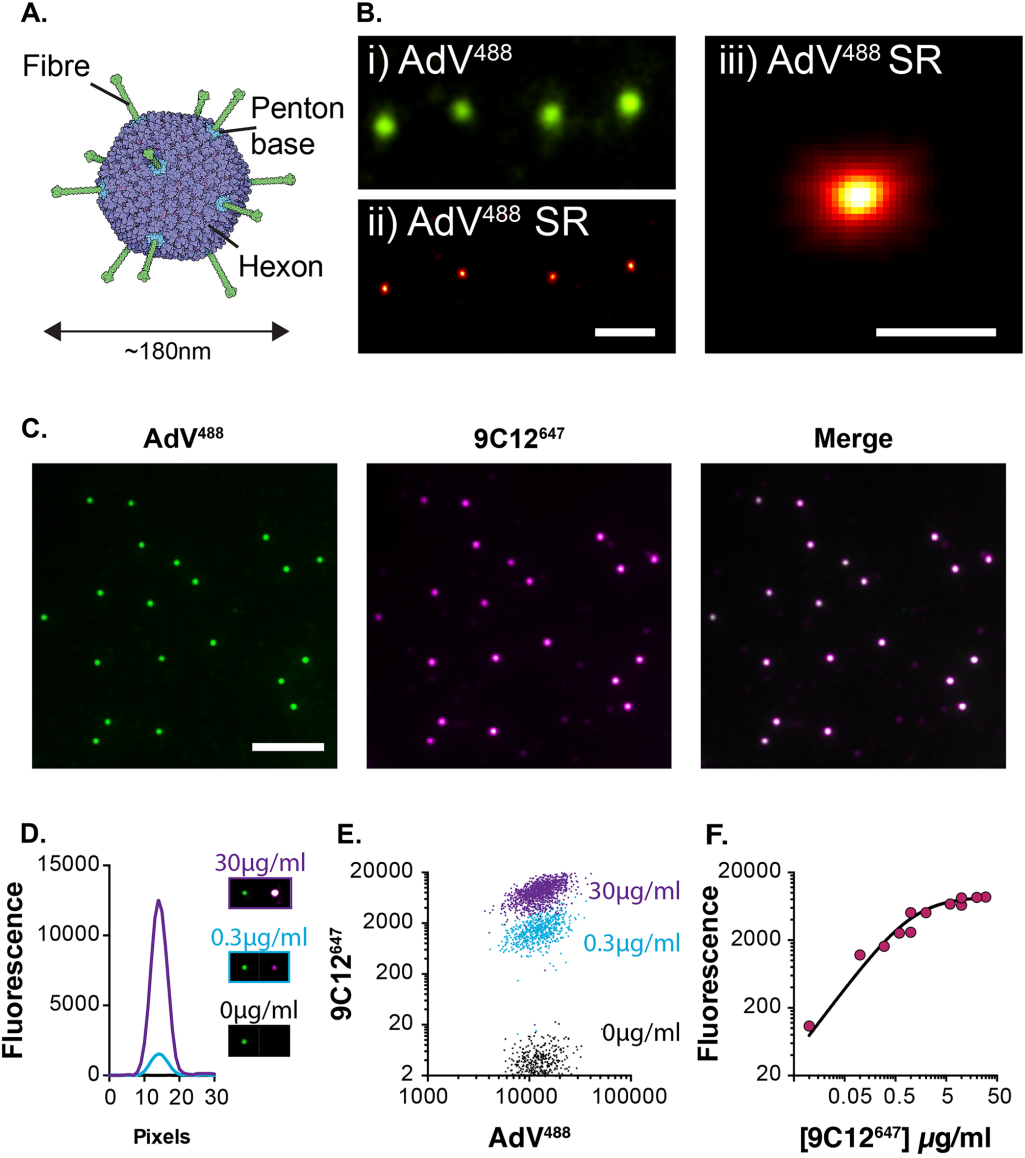
Experimental measurements of AdV-antibody complexes. (**A**) A molecular cartoon of an AdV particle; Ab 9C12 targets the hexon protein. (**B**) Immobilised AdV^488^ particles were imaged by TIRF-M (i) and SRRF (ii), iii provides an enlarged image of a super-resolved particle. Scale bars 1 $\upmu \hbox {m}$ (i & ii) and 200nm (iii). (**C**) A representative field of AdV^488^ particles incubated with 1$\upmu$g/ml 9C12^647^, the merge image displays an overlay of both channels. Scale bar 5$\upmu$m. (**D**) 9C12^647^ fluorescent signals associated with AdV particles incubated with 30, 0.3 and 0$\upmu$g/ml antibody. (**E**) Fluorescent measurements of populations of AdV particles. (**F**) Mean 9C12^647^ signals upon increasing concentration of antibody.

Successful implementation of our strategy requires sensitive and unambiguous measurements of individual virus-antibody complexes. We achieved this by immobilising AdV particles onto coverslips for analysis by total internal reflection fluorescence microscopy (TIRF-M; a detailed description of experimental methodology and technical notes are provided in the supplementary information). Prior to immobilisation, purified AdV particles were directly labelled with Alexa Fluor 488, this provided a reference label by which AdV particles could be unambiguously identified. When observed by TIRF-M (Fig. 3Bi) AdV^488^ appear as monodisperse diffraction limited spots (the particle being ${\sim }$180 nm in diameter; Fig. 3A). To confirm a monodisperse population we used SRRF analysis (super-resolution by radial fluctuations^20^); for each spot we resolved a single maxima of fluorescence that was ${\sim }$200 nm in diameter (Fig. 3Bii and iii), consistent with the ultrastructure of AdV particles (Fig. 3A).

Immobilised AdV particles were incubated with monoclonal antibody 9C12 conjugated to Alexa Fluor 647 dye (9C12^647^), and imaged by TIRF-M. Each AdV particle was positive for antibody, indicating the assembly of virus-antibody complexes (Fig. 3C). Moreover, individual AdV-9C12^647^ complexes could be analysed in a quantitative manner over a >100 fold range in antibody concentration (Fig. 3D). We automated this process to allow measurements of whole populations of virus particles at varying concentrations of antibody (Fig. 3E & F). 9C12^647^ signal intensity was proportional to antibody concentration and reached a plateau at high values; this indicates increasing virus-antibody stoichiometries up to a saturation point at which maximum antibody binding is achieved (as outlined in Fig. 1A). Moreover, the populations of virus particles were quite homogenous in 9C12 signal; this suggests a relative uniformity of assembly. In conclusion, we were able to quantitatively analyse the assembly of individual virus-antibody complexes.

**Figure 4:**
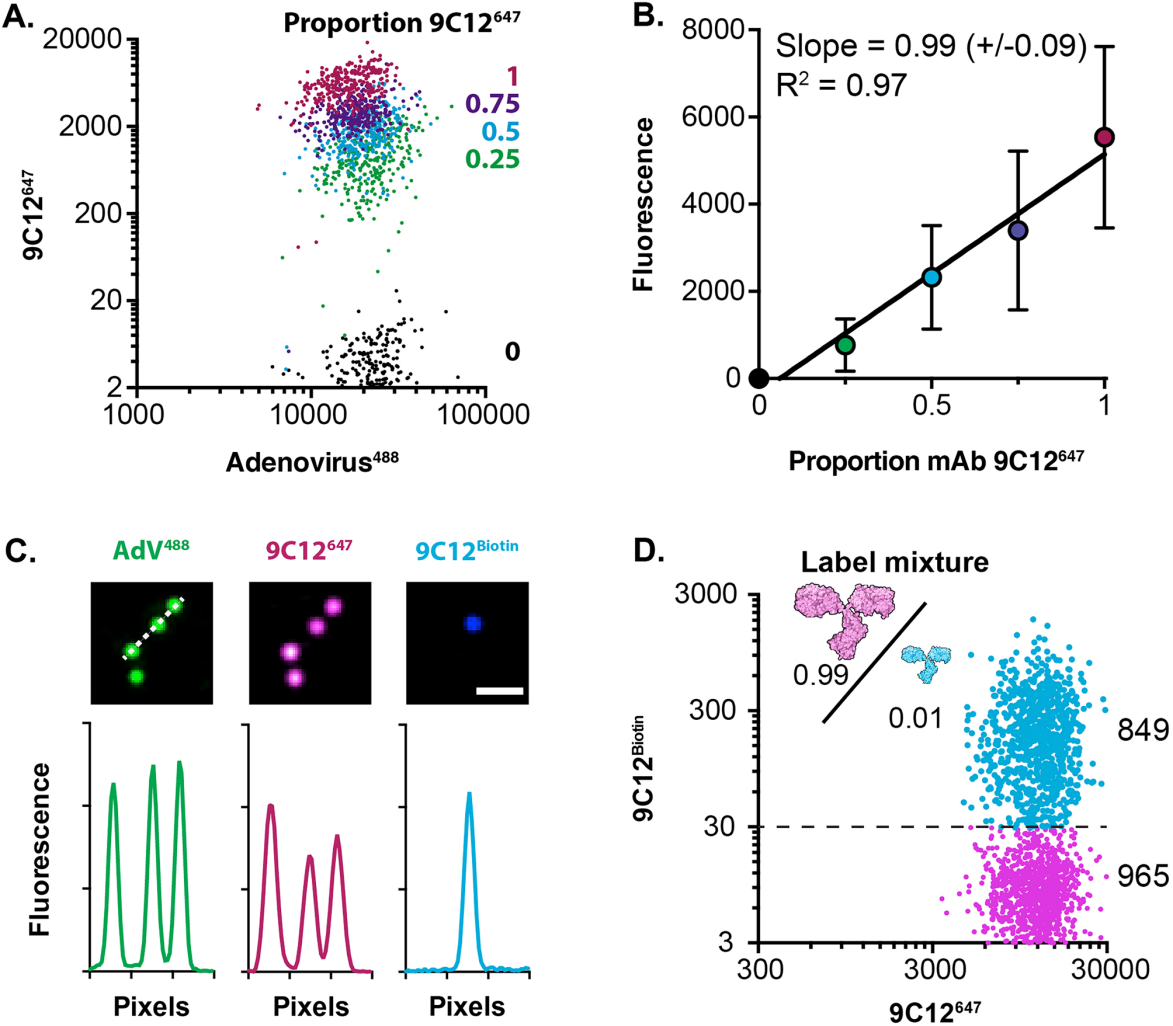
Binary labelling of AdV-antibody complexes. (**A**) AdV particles were incubated with mixtures of mAb 9C12 conjugated to either Alexa Fluor 647 (Ab^A^) or Biotin (Ab^B^), to a final concentration of 20$\upmu$g/ml. The scatter plot displays AdV^488^ and Ab^A^ fluorescent signals for populations of AdV particles incubated with decreasing proportions of Ab^A^. Data points are color-coded as stated in the legend. (**B**) The mean Ab^A^ (9C12^647^) signal has a near-perfect linear relationship to the proportion of Ab^A^ included in the antibody mixture, this indicates equivalent binding by Ab^A^ and Ab^B^. The linear regression slope and goodness of fit (R^2^) are provided (**C**) AdV^488^ particles (green) were incubated with 5$\upmu$g/ml 9C12^647^ (Ab^A^, magenta) spiked with 1% (0.01) 9C12^Biotin^ (Ab^B^, blue); only a minority of particles received any 9C12^Biotin^. (**D**) Scatter plot displaying a population of AdV particles (labelled as in C), which can be scored as being positive or negative for Ab^B^ (9C12^Biotin^), as annotated on the plot.

To achieve differential labelling a second batch of 9C12 was directly conjugated with biotin; this served as the Ab^B^ batch to be mixed with the 9C12^647^ Ab^A^ batch (Fig. 1). It may be possible that conjugation with either biotin or Alexa Fluor 647 has unexpected detrimental effects on antibody binding; therefore, to have confidence in our binary labelling system we needed to demonstrate fair competition between our differentially labelled antibody batches. To test this we incubated immobilised AdV particles with a high concentration of antibody (20$\upmu$g/ml) composed of different proportions of 9C12^647^ (Ab^A^) or 9C12^Biotin^ (Ab^B^) (e.g. 0.75:0.25, 0.5:0.5). We then monitored the 9C12^647^ (Ab^A^) fluorescence signal under each condition. As the proportion of 9C12^647^ (Ab^A^) dropped we measured a stepwise reduction in fluorescence signal (Fig. 4A). If both batches of antibody possess equivalent binding to AdV we would expect a linear relationship between the proportion 9C12^647^ and fluorescent signal. Indeed, when normalised for units, we observed a near perfect linear relationship (Fig. 4B, slope = 0.99, R^2^=0.97); indicating a fair competition in binding between Ab^A^ and Ab^B^.

As depicted in Fig. 1B, our approach requires detection of single antibody molecules of Ab^B^ within individual virus-antibody complexes. To explore this we incubated immobilised AdV particles with 5$\upmu$g/ml 9C12^647^ (Ab^A^) spiked with 1% 9C12^Biotin^ (Ab^B^) (i.e. f_l_ = 0.01). Molecules of 9C12^Biotin^ were detected using streptavidin (an ultra-high affinity biotin binding protein) conjugated to a quantum dot (QDot^655^). The photostability of quantum dots permits signal accumulation over prolonged exposure times^21,22^, therefore increasing the sensitivity of detection. Analysis by TIRF-M revealed that whilst every particle was positive for 9C12^647^ (Ab^A^) only a subset possessed 9C12^Biotin^-QDot^655^ (Ab^B^) signal (Fig. 4C); this suggests a population of AdV particles receiving one, or very few, Ab^B^ molecules (as outline in Fig. 1B). Automated analysis revealed well-separated populations of AdV particles that were positive or negative for 9C12^Biotin^ (Ab^B^) (Fig. 4D). Note that all particles were positive for 9C12^647^ (Ab^A^). These data are consistent with the assembly of virus-antibody complexes in which the vast majority of antibody molecules are from batch A (9C12^647^), but a subset of complexes contain $\sim$1 molecules of batch B antibody (9C12^Biotin^); the proportion of Ab^B^ positive particles will serve as the output data for statistical modelling.

Notably, our method is dependent on a precisely known value for $f_l$ and, therefore, it is important to demonstrate its robustness to experimental pipetting error when preparing the Ab^A^/Ab^B^ mixture. Typically, these mixtures were prepared using a 10$\mu$l pipette, which, based on the manufacturer’s data, can have a systematic error of $\le$2.5% (i.e. consistently inaccurate by + or − 2.5%) and a random error of +/−1.2% (i.e. stochastic error upon each pipetting motion). We, therefore, simulated the effect of this error on the accuracy of our statistical approach (Supplementary Fig. 1). Here, our model performed well despite experimental error, giving bias and standard deviation values equivalent to simulations without error (Fig. 2). Therefore, our method is robust to the level of inaccuracy typically introduced through pipetting error.

## Results

We proceeded with a series of experiments to generate data for statistical modelling and stoichiometric estimates. To achieve this we performed four independent overlapping titrations over a >100 fold range in antibody concentration (0.15–20$\upmu$g/ml); this range covers the saturation point, identified in Fig. 3F, and lower antibody concentrations that are biologically relevant for 9C12-mediated neutralisation of AdV^16^. Guided by the simulations in Fig. 2, we explored a range of Ab^B^ proportions, from 0.007 to 0.07 (0.7–7$\%$ ), and, where possible, collected >500 particles per sample (the average number of particles collected was >1500). The proportion of positive particles was assessed for each sample, details of data analysis are provided in the supplementary information.

Supplementary Fig. 2 provides representative data: scatter plots display Ab^A^ (9C12^647^) and Ab^B^ (9C12^Biotin^) intensities for control samples (treated with unmixed Ab^B^ or Ab^A^) and four representative test samples incubated with a titration of 9C12, containing 0.7% Ab^B^ ($f_l$=0.007). Bar charts provide summary statistics for each channel: the particles have uniform AdV^488^ reference signal, whereas the Ab^A^ fluorescence decreases with antibody concentration; likewise, the proportion of Ab^B^ positive particles decreases with concentration. These data are consistent with the expected concentration-dependent stepwise reduction in virus-antibody stoichiometry.

**Figure 5:**
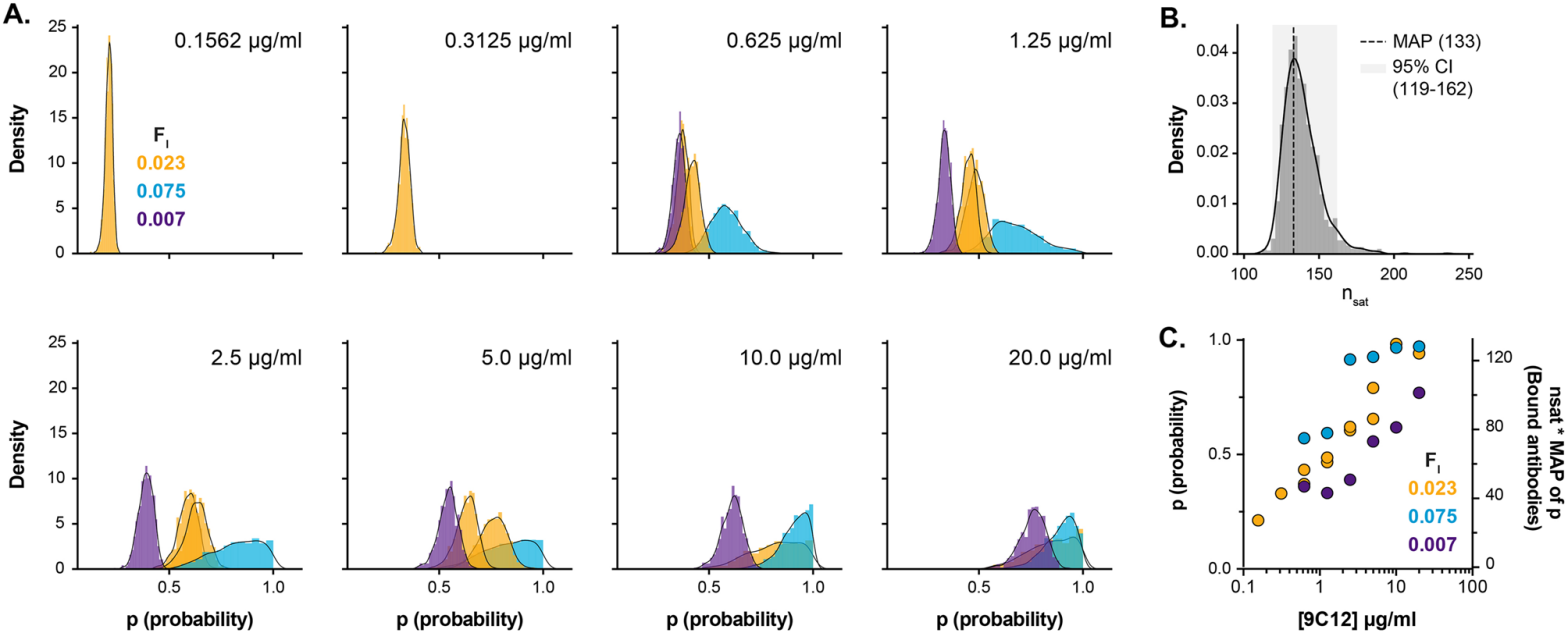
Posterior distributions and estimated parameters of AdV-9C12 interaction. Multiple titrations of Ab^A^ (mixed with f_l_=0.007-0.075 Ab^B^) were performed to allow statistical modelling of AdV-9C12 interaction stoichiometries. (**A**) Posteriors of antibody binding probabilities (p) for all measurements listed in Supplementary Table 1, grouped and color-coded by the proportion of Ab^B^ used in each experiment ($f_l$). (**B**) Posterior distribution of n_sat_, the number of antibodies bound to a virus at saturation. The maximum a posteriori (MAP) estimate of nsat is 133 and lies within a 95% credible interval of 119 to 162. (**C**) Scatter plot displaying MAP estimates of all binding probabilities inferred from the posterior distributions shown in A, plotted against antibody concentration. The axis on the right additionally shows the expected number of bound antibodies (n_sat_ * MAP of p).

**Figure 6:**
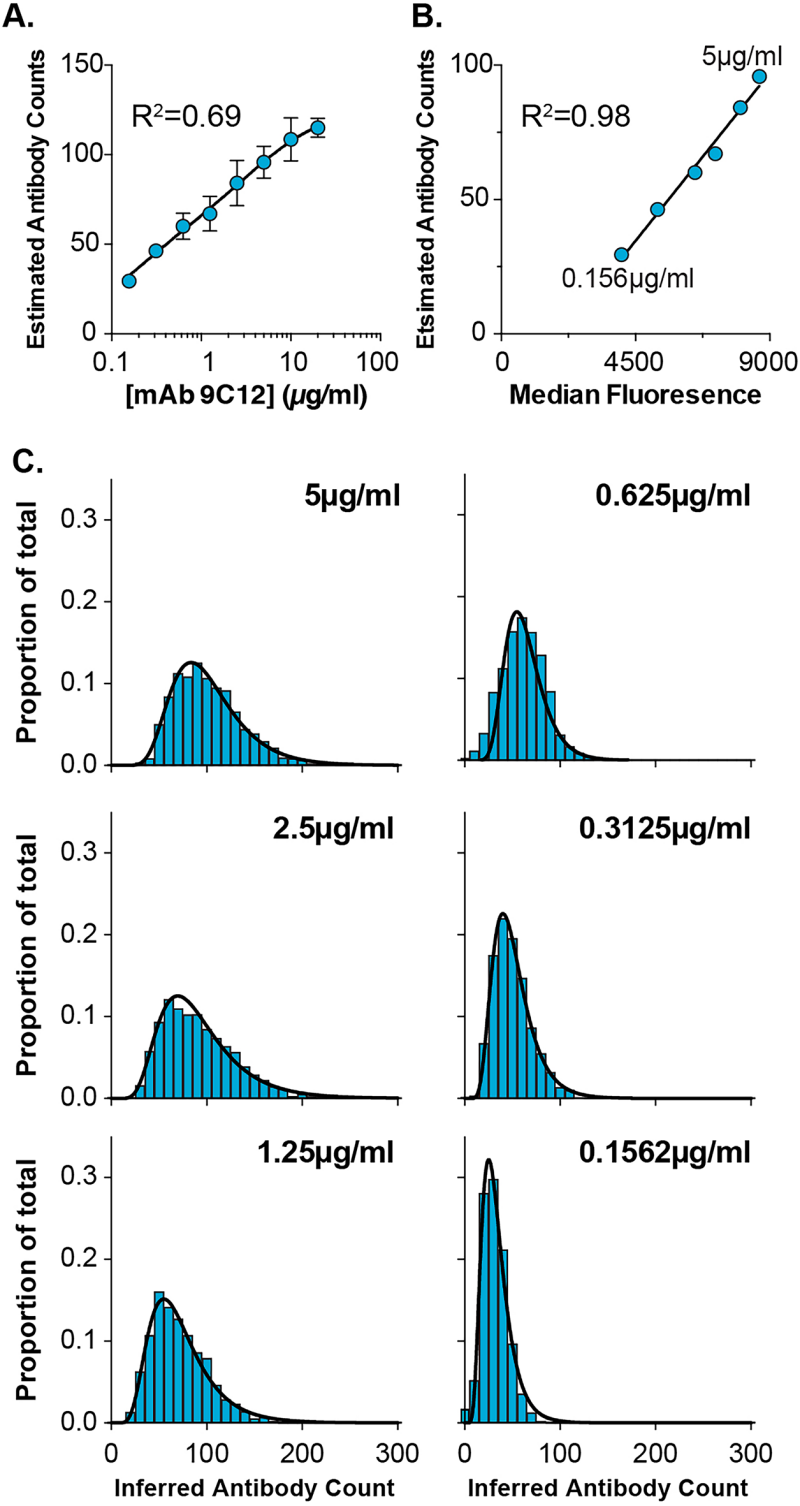
Inferring population heterogeneity. (**A**) Mean stoichiometric estimates at increasing concentrations of 9C12, error bars indicate standard error of the mean; data fitted with a binding curve, R^2^= 0.69 (Graphpad, Prism). (**B**) Stoichiometric estimates were compared to 9C12^647^ (Ab^A^) fluorescent values from an individual experiment; data fitted with a linear regression, R^2^= 0.98 (Graphpad, Prism). (**C**) Stoichiometric estimates were used to calibrate fluorescent intensity values allowing inference of heterogeneity. Histograms display the frequency of inferred antibody counts as a proportion of total particles. The frequency data were fitted with a log-normal distribution, R^2^ values were all >0.99 (Graphpad, Prism).

The four experiments generated 24 sets of binary scores (Supplementary Table 1), which were inputted to our statistical model, as outlined in the methods. This generated posterior distributions of p (probability of an antibody binding site being occupied) for each sample (Fig. 5A) and an estimated n_sat_ of 133 molecules (maximum a posteriori (MAP) estimate; credible interval [119, 162]; Fig. 5B). Absolute antibody numbers for each sample can be derived by multiplying the MAP estimate of each posterior distribution (for p) by n_sat_ (Fig. 5C). Figure 6A displays the mean number of bound antibodies at increasing 9C12 concentrations (derived from Fig. 5C); AdV-9C12 stoichiometries range from 29 to 115 across the titration of antibody. For any given sample, alongside the proportion of particles that are positive for Ab^B^, the experimental setup also provides fluorescent intensity values of Ab^A^ in each AdV-Ab complex (Supplementary Fig. 2); this provides an internal reference for AdV-9C12 interaction, therefore, the stoichiometric estimates should correlate with their experimentally-matched fluorescent intensity values. Figure 6B demonstrates a near-perfect linear correlation between stoichiometry and fluorescence intensities for an example experiment; this would suggest that our statistical modelling faithfully reports the relationship between antibody concentration and AdV binding occupancy.

The stoichiometric estimates generated by our method are derived from an ensemble measurement and, therefore, represent the antibody interactions of the average virus particle; this obscures heterogeneity within the population. However, given the excellent agreement between the estimated antibody counts and Ab^A^ (9C12^647^) intensity values (Fig. 6B) we used the stoichiometric estimates to calibrate the Ab^A^ fluorescence signals, therefore allowing us to infer population heterogeneity. To achieve this, for any given sample we matched the median Ab^A^ fluorescence intensity to its associated stoichiometic estimate (generated by the model); extrapolating from this we then converted the Ab^A^ fluorescence values to inferred antibody counts for individual virus particles. Figure 6C provides histograms and frequency distributions of inferred antibody counts for a range of concentrations from a single experiment (as shown in Fig. 6B). Being slightly skewed to the right, the frequency data was best fitted using a log-normal distribution, this is particularly apparent at higher antibody concentrations. This would suggest that a significant proportion of virus particles are binding more antibodies than the average particle. However, no particles bound greater than 230 antibody molecules. We provide an interpretation of these observations in the discussion.

In summary, we have used statistical modelling to derive stoichiometric estimates of AdV-9C12 complexes. This suggests that the most probable antibody binding maximum is, on average, 133 molecules (95% CI 119-162). However, using stoichiometric estimates to calibrate fluorescent data revealed population heterogeneity with a small proportion of virus particles binding ${\sim }$200 antibody molecules. Notably, these values are in excellent agreement with previous estimates that we, and others, have derived using alternative methods^16,17^.

AdV + 9C12 is a well-understood model system and, therefore, is ideal to develop and validate our molecular counting method. However, the technical and analytical framework, outlined here, will be generally applicable to other molecular complexes. Achieving reliable estimates requires the appropriate choice of $f_l$ (the proportion of ’B’-labelled component); in the case of AdV + 9C12 this could be guided by previous estimates of the stoichiometry at complete saturation (Fig. 2). For many bio-molecular assemblies $n_{\text {sat}}$ is poorly defined, or completely unknown, therefore to evaluate the utility of our method under such circumstances we calculated the Fisher information for theoretical complexes with $n_{\text {sat}}$ values ranging from 10-1000. This analysis is provided in Supplementary Fig. 3 with data expressed as inverse Fisher information, where low values indicate optimal conditions under which reliable estimates can be achieved. As $n_{\text {sat}}$ increases there are fewer experimental conditions that reach optimum performance, nonetheless, low $f_l$ values are likely to give accurate molecular counts over a very broad range of $n_{\text {sat}}$ stoichiometries. To test this further, informed by the Fisher information analysis we picked a constant $f_l$ value of 0.01 and simulated experiments at $n_{\text {sat}}$ values of 10, 50 and 100 (analogous to the simulations in Fig. 2, where $n_{\text {sat}}$=205). In these simulations our method performed well (i.e. low bias and standard deviation) across all $n_{\text {sat}}$ values, albeit with a necessity to sample greater numbers of virus-antibody complexes (V) at lower $n_{\text {sat}}$ values (Supplementary Fig. 4). This analysis suggests that our method is suitable for the quantification of bio-molecular assemblies with a wide range of stoichiometries.

## Discussion

Various methods permit the investigation of molecular stoichiometries within biological assemblies but technical limitations often make it difficult to obtain reliable estimates. For example SMLM, by its very nature, identifies single molecules and, if properly calibrated, can deliver accurate stoichiometries; however, successful counting by SMLM requires a very detailed understanding of the photochemical behaviour of the chosen fluorescent dyes. Here we outline a robust, and relatively easy, experimental framework for extracting accurate molecular counts using (non-SMLM) fluorescent microscopy and statistical modelling.

By creating a scheme in which complexes need only be qualitatively scored for a particular label, our method negates the necessity for carefully calibrated measurements and an *a priori* understanding of the system. The only requirement is that the chosen label is clearly discerned from background; this is easily achievable with bright/stable fluorescent dyes and relatively inexpensive cameras; in this case we used quantum dots for sensitive detection, but many standard fluorescent dyes should also suffice.

An obvious limitation is that our method relies on an ensemble measurement and obscures heterogeneity within the population of complexes. However, this information remains accessible via the A-label fluorescent signals measured from each complex. Consequently, the ensemble-based stoichiometric estimates of the average complex can be used to calibrate these fluorescent signals and infer approximate molecular counts for individual complexes, therefore, restoring heterogeneity.

We were able to make robust measurements of AdV-9C12 interactions by fluorescence microscopy and successfully implemented the differential labelling strategy. We performed multiple independent measurements at various antibody concentrations to derive molecular counts for AdV-9C12 complexes. Our analysis estimated that the average AdV particle interacts with a maximum of 133 9C12 antibody molecules. Moreover, through examination of population heterogeneity we revealed that a small proportion of AdV particles may bind up to 230 antibody molecules.

These data can be reconciled with a molecular model of AdV-9C12 complexes. AdV particles possess 720 identical hexon subunits, each providing a potential binding site for 9C12, however, previous estimates suggest an absolute maximum of 240 antibody molecules per virion. This would suggest that particle geometry places packing constraints on the arrangement of antibody molecules. Cryo-EM analyses indicates a complex and heterogeneous interaction network in which particle geometry creates potential antibody clashes and, therefore, prevents binding to every site simultaneously^17^. Whilst there are consistently five 9C12 molecules at each of the twelve vertices of the AdV particle, additional antibody interactions occur through heterogeneous packing across the surface; the pattern of which is likely dictated by the random order in which binding sites become occupied on any given virus particle. Consequently, with optimal antibody packing there is likely to be an absolute maximum binding occupancy of $\sim$240 molecules. Our data indicate that the average particle binds fewer molecules (133) than the threshold dictated by purely geometric limitations. This would suggest that the majority of particles do not achieve optimal antibody packing and saturate at lower occupancies. This model also offers explanation to our observation that no particles bind greater than $\sim$230 molecules (Fig. 6C). Alternatively, the observed occupancy may be influenced by the inability of antibodies to access binding sites that are closely apposed to the coverslip surface; although, the penton fibre ’spikes’ of Adenovirus particles are likely to provide sufficient elevation from the coverslip to permit antibody binding. Nonetheless, binding site accessibility needs to be considered when applying our method to other systems.

These interpretations expose a potential flaw in our approach. Our modelling strategy assumes that components bind independently, but in this test case the geometry of AdV particles create clashes between adjacent 9C12 molecules such that complete saturation is not possible. Consequently, antibody binding events can be influenced by prior antibody interactions and, therefore, are not independent. Although this may have introduced inaccuracies in our estimates, the molecular counts derived from our approach are in good agreement with previous values. Moreover, we maintain that the assumption of independent binding is appropriate for a generalisable method that can be applied to other biological assemblies.

Calculating the Fisher information of our method demonstrates that it is capable of reliable quantification over a broad range of $n_{\text {sat}}$ stoichiometries (we tested 10-1000; Supplementary Fig. 3), and this was supported by further simulated experiments at low $n_{\text {sat}}$ values (Supplementary Fig. 4). These analyses can be used to guide experiments with other biological systems. Moreover, in the absence of any *a priori* knowledge on maximum stoichiometries we recommend using low $f_l$ (i.e. very little ’B’-labelled component), which will perform well across a wide range of $n_{\text {sat}}$. Given this, we expect our method to have broad utility. For example, within virology, investigating the molecular composition and antibody-mediated neutralisation of enveloped viruses such as human immunodeficiency virus, hepatitis C virus and SARS-coronavirus-2 (probable $n_{\text {sat}}$ values<50). Beyond the confines of virology, our method could be applied to a variety of other biological assemblies of various scales, for example: bacteria; purified cellular organelles; cellular vesicles, such as exosomes; and supramolecular complexes such as ribosomes or inflammasomes. Moreover, we expect our method could be integrated with other complementary methods to enhance quantitative analysis; for example it may provide a means of internally calibrating SMLM-based counting schemes.

In conclusion, we have developed a novel and robust method for counting components within biomolecular complexes. We demonstrate that this approach can provide accurate counts and could be applied in a wide range of other biological systems.

## Supplementary Information

Supplementary Information 1.

Supplementary Information 2.
