# Supplementary material for: A novel and robust method for counting components within bio-molecular complexes using fluorescence microscopy and statistical modelling
Source: Sci Rep. 2022 Oct 14;12:17286. doi: 10.1038/s41598-022-20506-y (PMC9568568; doi:10.1038/s41598-022-20506-y)
Supplement: Supplementary file 1 — Supplementary Information 1. [file 41598_2022_20506_MOESM1_ESM.pdf]

Supplementary Figures for: **A novel and robust method for counting components within bio-molecular complexes using fluorescence microscopy and statistical modelling.**

Mersmann et. al.

	Experiment 1		Experiment 2		Experiment 3		Experiment 4	
	f_l = 0.075		f_l = 0.0075		f_l = 0.023		f_l = 0.023	
**[9C12] μg/ml**	**V**	**Ab^B positive**	**V**	**Ab^B positive**	**V**	**Ab^B positive**	**V**	**Ab^B positive**
**20**	1190	1	3143	0.545020681	427	0.941451991		
**10**	648	1	3070	0.46970684	839	0.94398093		
**5**	900	1	1976	0.430161943	1072	0.906716418	1160	0.912068966
**2.5**	862	1	1632	0.330882353	1414	0.859971711	2541	0.866587957
**1.25**	799	0.998748436	2258	0.291851196	1003	0.77666999	1332	0.795045045
**0.625**	1254	0.997607656	3117	0.306705165	725	0.728275862	1840	0.70326087
**0.3125**							1793	0.658672616
**0.15625**							4237	0.47911258

[9C12] μg/ml = total concentration of antibody
V = number of virus particles analysed
Ab^B positive = proportion of particles scoring positive
f_l = proportion of Ab^B included in the experiment

Supplementary Table 1. Outputs used for statistical modeling of virus-antibody stoichiometries. We performed four independent overlapping antibody titrations at varying f_l values.

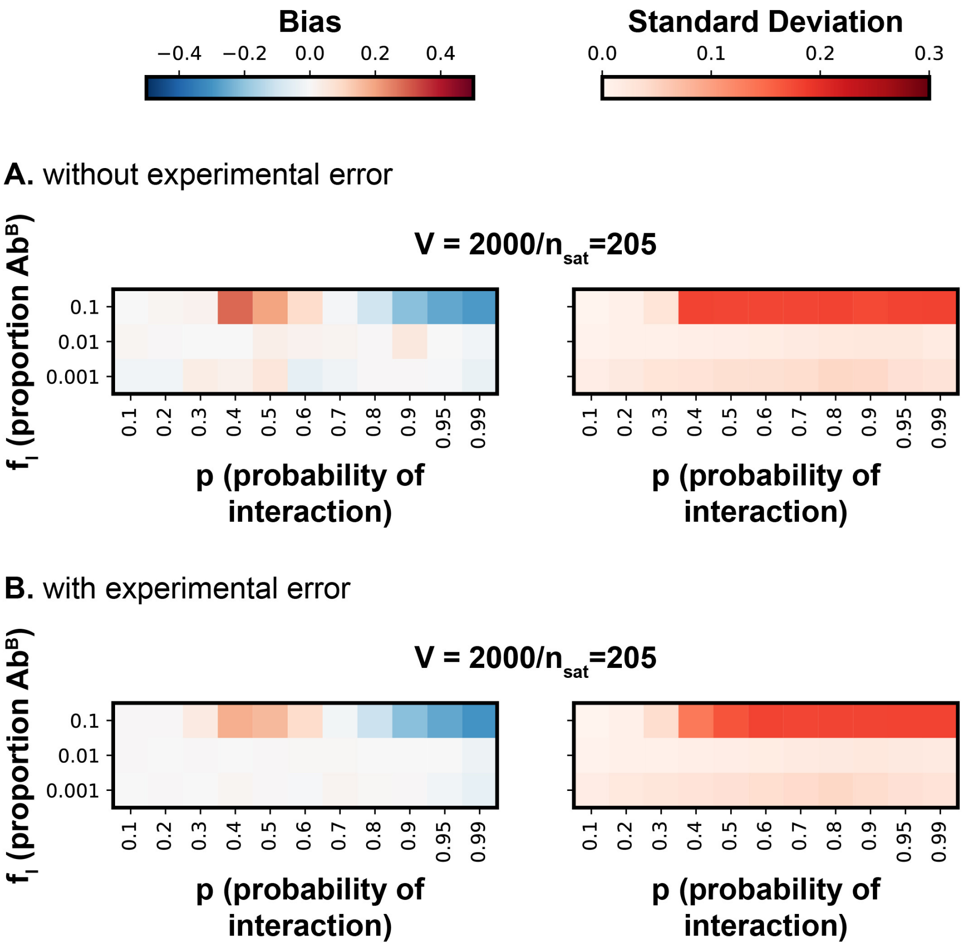

Supplementary Figure 1. Modelling the effect of experimental error. Bias and standard deviation landscapes of the posterior distributions, estimated from simulations to explore the effect of experimental error on f_l. **A.** Bias and standard deviation without experimental error (as in Figure 2.). **B.** Bias and standard deviation with the introduction of experimental error in f_l. Here we modelled pipetting error informed by manufacturer's data: with systematic error (i.e. occurring on every pipetting motion) of 2.5% and a random error (i.e. occurring stochastically with each pipetting motion) of +/- 1.2%. In all cases f_l was varied over a 100 fold range, with constant V=2000 and n_{sat}=205 (analogous to the analysis in Figure 2.).

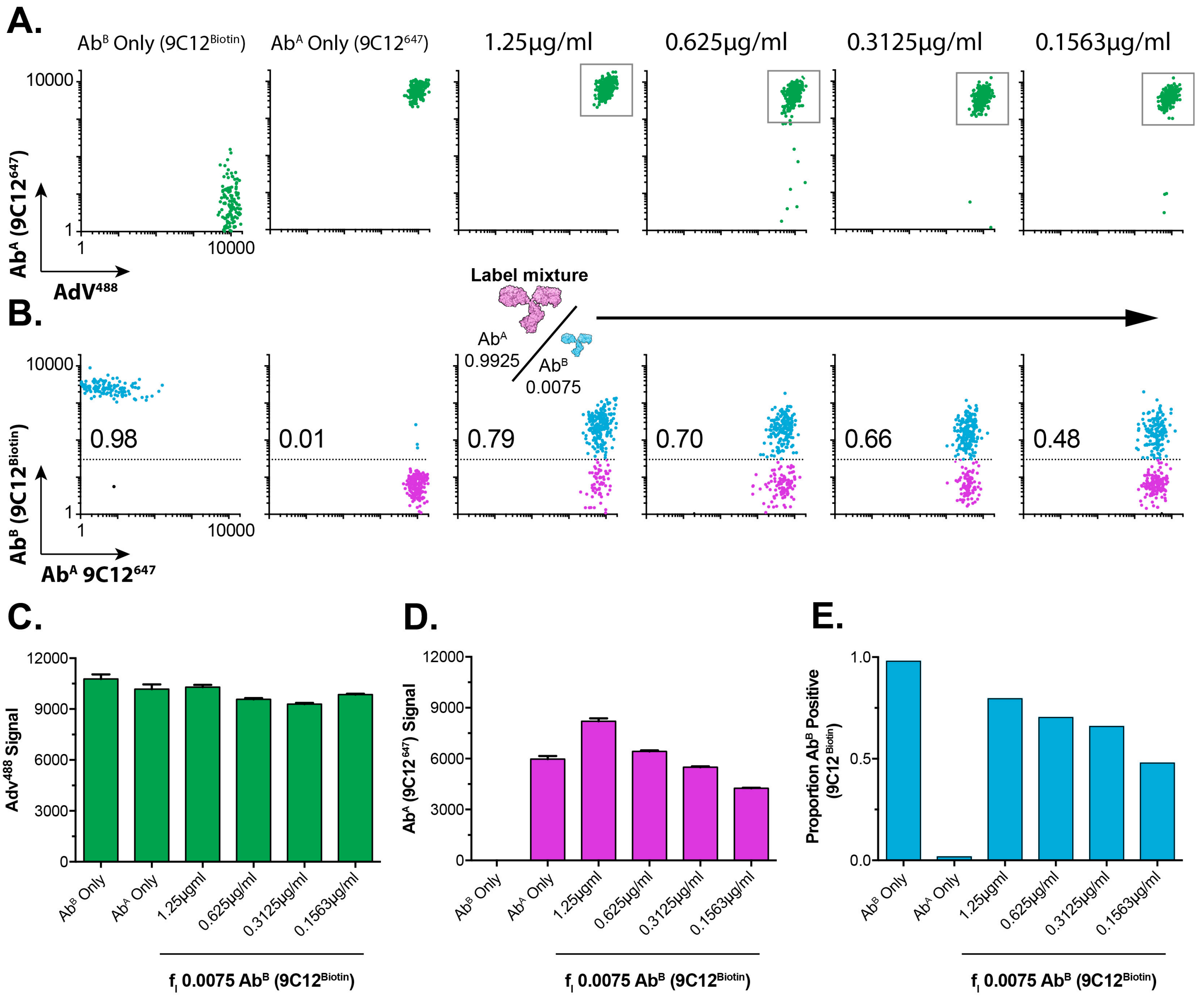

Supplementary Figure 2. Quantification of differentially labelled virus-antibody complexes.

A. Scatter plot, where each data point represent a measurement from an individual virus particle, displaying AdV^{488} and Ab^{A} ($9C12^{647}$) signals. For clarity only 300 randomly selected particles are shown in each plot. Control samples incubated with Ab^{B} ($9C12^{Biotin}$) or Ab^{A} ($9C12^{647}$) alone at 0.625µg/ml, are on the left. Plots to the right display particles incubated with the stated concentrations of 9C12 with f_I=0.007 Ab^{B}. Downstream analysis of Ab^{B} ($9C12^{Biotin}$) was performed only on particles with Ab^{A} signal (boxed particles) **B.** Scatter plots displaying Ab^{A} and Ab^{B} signals for the same 300 virus particles. Ab^{B} positive particles (signal ≥30, dotted line) are color coded in blue, the proportion of positive particles is annotated for each sample. **C.** The mean AdV^{488} fluorescent intensity signals for each sample; ~2000 particles were analysed in each condition. **D.** The mean Ab^{A} fluorescent intensity signals for each sample. **F.** The proportion of Ab^{B} positive particles for each sample. Error bars indicate standard error of the mean.

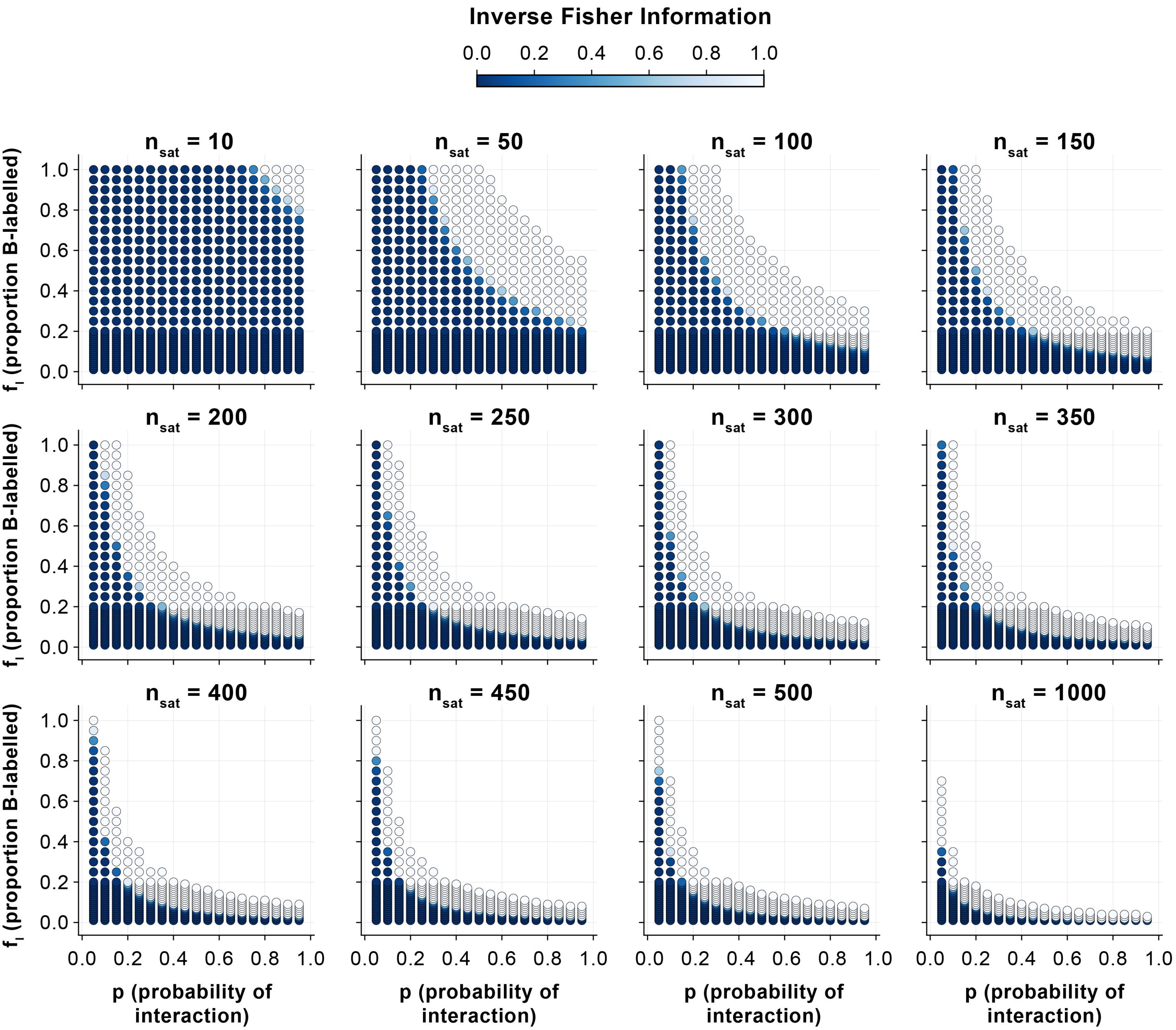

Supplementary Figure 3. Inverse Fisher information under varying values of n_{sat}. To aide general application of our approach we calculated the Fisher information for theoretical protein complexes with n_{sat} stoichiometries of between 10-1000 (one per plot, as annotated). Each plot provides data points color-coded for inverse Fisher information (as denoted in the key) upon varying f_l (proportion of ‘B’-labelled component) and p (probability of binding) with 2000 complexes being analysed for each. Fisher information reflects the ability of an experimental setup to extract reliable estimates; low values (i.e. dark blue data points) represent optimal conditions. For a broad range of n_{sat} stoichiometries, low fl values (0.001-0.1) are likely to achieve accurate molecular count estimates. Areas with no data points indicate conditions that are unlikely to yield reliable estimates.

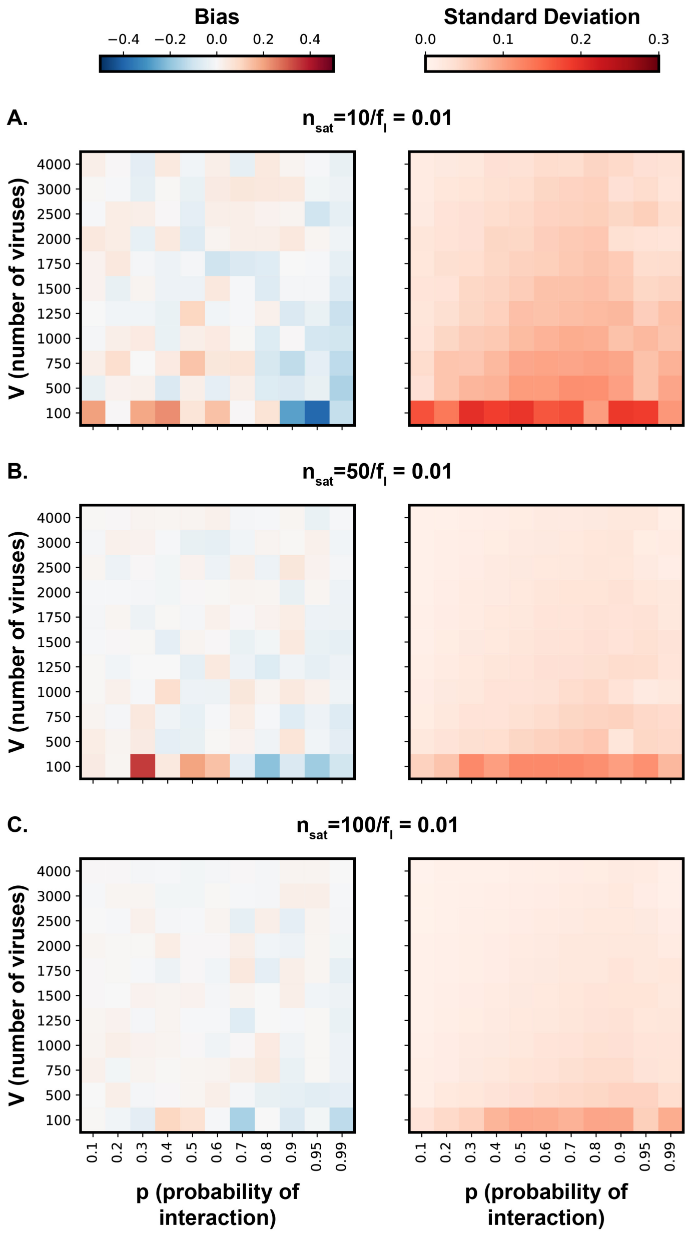

Supplementary Figure 4. Model verification under varying n_{sat}. Bias and standard deviation landscapes of the posterior distributions, estimated from simulations to explore varying n_{sat} values. Here, bias is the mean of the posterior distribution minus the true probability, while standard deviation refers to the standard deviation of the posterior distribution. n_{sat} values of 10 (**A.**), 50 (**B.**) and 100 (**C.**) were simulated under varying V (number of viruses/complexes) and p (probability of interaction), in each case n_{sat} was given an arbitrarily high upper bound of 1000. Informed by Fisher information analysis (Supplementary Figure 3), f_l was set at 0.01 in each case.
