# Supplementary material for: A novel and robust method for counting components within bio-molecular complexes using fluorescence microscopy and statistical modelling
Source: Sci Rep. 2022 Oct 14;12:17286. doi: 10.1038/s41598-022-20506-y (PMC9568568; doi:10.1038/s41598-022-20506-y)
Supplement: Supplementary file 2 — Supplementary Information 2. [file 41598_2022_20506_MOESM2_ESM.pdf]

Supplementary Methods for: **A novel and robust method for counting components within bio-molecular complexes using fluorescence microscopy and statistical modelling.**

Mersmann et.al.

Virus preparations

Replication-deficient human adenovirus-5 stocks were generated in a transcomplementation 293F cell line for 72 hours and purified by two rounds of CsCl centrifugation. Purified particles were conjugated to Alexa Fluor 488 using a protein labelling kit (Thermo Fisher Scientific).

Antibody production and labelling

9C12 anti-hexon monoclonal antibody was purified from hybridoma supernatants on a protein G column (GE Healthcare). Protein labelling kits were used to conjugate 'A' batch antibody to Alexa Fluor 647 (Thermo Fisher Scientific) and 'B' batch antibody to biotin (Merck Life Sciences). The degree of biotinylation was determined using a fluorescence biotin quantitation kit (Thermo Fisher Scientific).

Note when choosing an A:B mixing ratio (f_l) for investigations of assemblies where little/no *a priori* knowledge is available (i.e. there is no reasonable estimate of n_{sat}) our analysis (Supplementary Figure 3) indicates that low f_l values (0.001-0.1) should perform well across a broad range of potential n_{sat} stoichiometries. For assemblies where n_{sat} can be approximated we recommend using Supplementary Figure 3 to choose appropriate f_l mixing ratios; conditions with dark blue data points are optimal.

Note that accurate determination of the degree of labelling for 'B' batch component (in this case biotin conjugation) is critical for achieving reliable molecular counts. Ideally the ratio should be >1 biotin/fluorophore label per molecule. Labelling ratios of <1 will need to be accounted for when determining molecular counts. Similarly, great care needs to be taken when mixing 'A' and 'B' labelled components to ensure an accurate f_l value and, therefore, accurate molecular counts.

Note it is important to ensure little/no spectral overlap between the chosen 'A' and 'B' labels, allowing good discrimination of positive and negative complexes. In this respect, the combination of A= Alexa Fluor 647 and B= biotin + QDot 655 provides excellent separation due to the short wavelength excitation of QDots.

Sample preparation

25mm coverslips received two 2-minute washes in each of the following ddH_20, ethanol and methanol. They were then treated with 2% 3-amino-propyltriethoxysilane (Merck Life Sciences), diluted in acetone, for 5 minutes before being rinsed twice in ddH_2O. Each slide was mounted in an Attofluor chamber (Thermo Fisher Scientific) to which AdV, diluted in PBS, was then added. The samples were incubated in the dark overnight in a humidified box at 4°C, with gentle shaking.

The following day, the samples were rinsed in PBS and then blocked for 30-minutes in 1% BSA (diluted in PBS and 0.2µm filtered). Following two PBS rinses the samples were then incubated for one hour with 9C12 antibody (at various concentrations and combinations of A and B batches, as stated in the text). Unbound antibody was washed away by two sequential rinses in PBS, the samples were then fixed for 15 minutes in 4% EM-grade formaldehyde (Thermo Fisher Scientific),

followed by two further PBS rinses. To detect biotinylated antibody, samples were incubated for 15-minutes with 2nM QDot 655 streptavidin conjugates, followed by two final PBS rinses. Samples were gently agitated on a rocking platform throughout the staining procedure. PBS was used as diluent at all stages.

Note, the photostability of QDots make them ideal for long exposure times, providing excellent signal. However, due to their size (~20nm), QDots can create steric clashes if used at high density. Therefore, we only recommend that they are used for the minority 'B' batch of labelled component, such that only a low number of QDots (~1) are present in any given complex.

Note We found air-drying to be very detrimental to sample preparation and therefore minimised the opportunity for this to occur throughout the entire sample preparation process. For example we used two 1000µl pipettes when rinsing coverslips; one to aspirate, one to immediately add fresh buffer.

Microscopy

Samples were imaged on a Nikon Ti inverted microscope (Nikon Instruments), through a 100X oil objective, with 405nm (QDot), 488nm (Alexa Fluor 488) and 635nm (Alexa Fluor 647) laser illumination in TIRF mode. Images were captured using a 1024x1024 region of interest on an ORCA-Flash 4.0 sCMOS camera (Hamamatsu).

Data Analysis

Images were processed in ImageJ using a custom image analysis macro, which includes the channel align tool from the NanoJ package (Ricardo Henriques, https://github.com/HenriquesLab). The analysis pipeline outputs fluorescent intensity values for each particle in all three channels; particles were scored positive for $9C12^{Biotin}$ (B label) if they had a signal >30 (2X standard deviations above background). Scoring was manually verified for a random selection of images. The raw imaging data used to derive molecular counts, and the ImageJ analysis macro, are provided here: 10.5281/zenodo.3955142.

Note it is not necessary to perform multiple independent titrations to execute the statistical modelling outline in this work. However, due to the inherent error and variability when making measurements of biological systems we recommend performing repeat experiments.
